# Surfing alone? The Internet and social capital: evidence from Indonesia

**DOI:** 10.1186/s40008-022-00267-7

**Published:** 2022-07-27

**Authors:** Bayu Kharisma

**Affiliations:** Department of Economics, Universitas Padjadjaran, Bandung, Indonesia

**Keywords:** Internet access, Social capital, Indonesian Family Life Survey (IFLS), Instrumental variable

## Abstract

The objective of this study is to determine how internet access affects social capital in Indonesia’s community, based on the data from the fifth wave of the Indonesian Family Life Survey (IFLS) with the instrumental variable (IV) method. The results showed that the use of the Internet plays a significant role in strengthening social capital in Indonesia, especially for male heads of households that have a relationship with neighbors with strong internal cohesion and mutual trust. Internet users in Java and Bali are more affectial in strengthening social capital compared to users outside the islands located in eastern Indonesia. This disparity is because most Indonesian internet users live in the western part of the country. Furthermore, there is a development disparity in regard to internet infrastructure and internet connection service availability throughout the country.

## Introduction

Nowadays, internet access has penetrated various aspects of our lives, for example, education, social, political, economic, cultural, and health. The Internet now plays a significant role in information exchange and is a medium and a bridge to communicate efficiently with no limitation of time and space. Additionally, the Internet makes all work easier and more efficient. It can also be used to expand knowledge and connect to other people as humans are social beings. The Internet has become customary in our everyday lives and is no longer a niche—almost everyone has felt the effects and benefits of the Internet no matter what they are doing. The Internet can be used to refresh the mind for entertainment, alleviates education access by easing information accessibility, opens doors for movies, music, and other media, and overall a great way to vent and disconnect from everyday fatigues and stress.

The Internet has been tied down into the threads of modernity and has become one of the leading players in education nowadays; people of all ages can now have better access to education—whether at home, while shopping, and of course, in schools. Internet access helps conduct any business anywhere globally—from just playing an online game to chatting and meeting serious clients from across the globe. However, some people believe that relationships in the real world might be neglected as virtual relationships become more important. In this case, as a mode of rapid communication, the Internet may free the individual from family ties and open opportunities to join new cultures and communities.

The speed and quality of information obtained through online media push the preferences of people to communicate via the Internet instead of conventional media. Zalnieriute (2013) argues that the Internet is a double-edged sword, supporting and sometimes detrimental to social relationships and mental health. Social media and networking sites can open the doors for connection with family, friends, strangers, and even celebrities, and they can help the users to manage and create new connections and allow for more freedom of thoughts, expression, and identity. Pénard and Poussing (2010) revealed that internet access helps communication, not only with family but also with strangers via e-mail, short messages, or social media platforms. In addition, the Internet has become a medium to enhance connections with families and friends and form new bonds and broader friendships. Internet access is often associated with relationships network, while social capital is commonly used to describe the quality and quantity of social interaction.

The beginning of the industrial revolution 4.0 has pushed new frontiers in terms of economic information and the overalls business day to day; consequently, society’s social fabric has been changed. With better quality of information technology, information transfer over distances will be easier and faster, increasing the intensity of long-distance social communication. However, a better quality of information technology may decrease face-to-face interactions (Sabatini and Sarracino 2017). This decrease may be because IT users tend to be more comfortable enjoying the advantages of IT development.

Information and communication technology are advancing at an unprecedented rate, and on a large scale. Experts call this phenomenon the "communication revolution." Samudhram (2010) stated that the communication revolution is the remarkable acceleration of communication technology, as seen in the increasing use of satellites, microprocessors, computers, and high-level radio services, and the changes that occur due to the alterations in the social, political, economic, and cultural aspects. In addition, the communication revolution is part of the human life history that is taking place at this moment.

**Figure.1:**
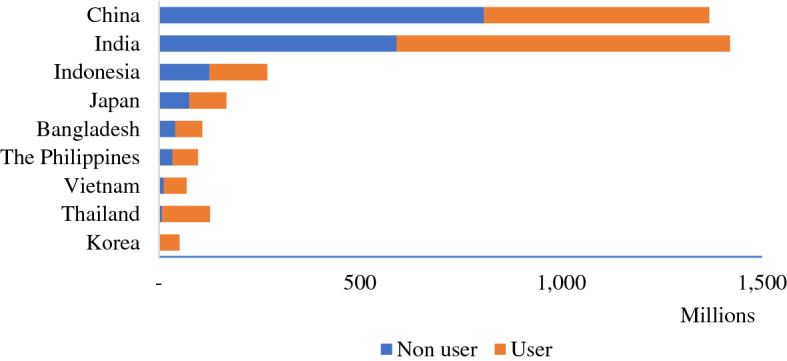
Internet users in Asia, 2019. Source: Internet World Stats, (2019)

From 2013 to 2018, Indonesia was ranked 6th globally among countries with the most access to internet (eMarketer 2014). Internet access in Indonesian has increased significantly, especially in the past 5 years, when it has increased by 50.2 million users. Based on Indonesia Investment (2019), Indonesian internet users in 2019 reached 184.97 million, increasing from 171.17 million in 2018 and 144.17 million in 2017. The majority of the users came from Java Island (55 percent). Meanwhile, internet usage in Indonesia has reached around 53 percent of the total population, which is 143.26 million of 269.54 million people. In short, internet users in Indonesia comprise 6.5 percent of Asia’s internet use, third behind China (829 million) and India (560 million) (Internet World Stats 2019). More details can be seen in Fig. 1.

Nurhayati-Wolff (2021) predicted that roughly 191 million Indonesians would utilize the Internet in 2020. This is anticipated to affect around 240 million people by 2025. Indonesia has over 171 million Internet users, making it one of the world’s largest online markets. In addition, by July 2021, online penetration in Indonesia will reach approximately 70%. YouTube is the most popular social network in Indonesia, with over 94% of the platform’s online population. Mobile internet connectivity is expanding at double-digit rates and accounts for more than 64 percent of the existing population.

Indonesia’s considerable number of internet users can indirectly affect social interaction, especially with the global COVID-19 pandemic. Social and physical distancing policies from the government require society to work, study, and worship from home, resulting in an increasing need for the Internet. Badan Pusat Statistik (2021) reported that 78.18% of households in Indonesia had used the Internet in 2020. This number increased by 4.43 points compared to 73.75% in the previous year. The increase in internet penetration is in line with the restrictions on community activities during the COVID-19 coronavirus pandemic. Various activities of working, studying, and shopping are mostly done by utilizing digital technology from home.

The Internet has become a forefront of discussion and cause for concern for academics, general society, and the government. How the Internet affects social connections has become a significant point of debate over the last decade. As previously mentioned, it can be a double-edged sword, where the public may see it as merely having benefits, whereas academic literature has been quick to point out that it can be detrimental to mental health as it bridges social isolation. When the Internet is only used passively, like a TV where the user receives the information, it can prevent social interaction from taking place.

Social capital is currently widely used by academics and practitioners in various studies. It is mainly present as an alternative form of modalities, such as economic capital, cultural capital, and human capital. Bourdieu (2018) introduced the concept of social capital during the debate over the forms of modality. He debated the forms by showing the opportunities for social capital to be converted. Bourdieu (2018) stated that not only economic capital can be converted into money but also cultural capital, which, in certain situations, can be converted into capital with economic value.

Putnam (2020) demonstrated that the decrease in social capital from fewer people participating in formal organizations, informal social connections, and trust among individuals in the US had been factored by a few things, including a) less available time for social interaction from labor flexibility and increased travel time; b) better access and mobility for laborers and students; c) development in technology. Additionally, TV and other entertainment media like multiplayer online games eventually replaced outdoor activities during an individual’s time. This point aligns with the argument that the Internet plays a role in social phobia and loneliness (Romano et al. 2016). It can fragment society into new virtual realities of new commonalities, negating the need for face-to-face connections (Antoci, Sabatini, & Sodini, 2012) or reducing the necessity for face-to-face meetings (Conrads & Reggiani 2017). Alternatively, Internet can also increase social interactions as it can help users overcome social anxiety and support the negative mood swings synonymous with loneliness (Clayton et al. 2013), increase online social participation (Duggan and Brenner 2013), and perhaps most significantly, help revive dormant old connections (Hampton, Goulet, Rainie, and Kristen 2011). Consequently, this implies that non-internet users may face issues with social integration. In this case, the lack of digital connection may exclude individuals from integrating into society, exacerbating social disparity and inequality. In short, individuals may have more social capital when they have internet access in this day and age.

Past research has reiterated how internet usage plays a crucial role in social capital in developing countries with mixed results. Siraj (2018) voiced that Pakistan’s internet usage has substantially reduced the need for telephone cable and face-to-face connection, increased the gap among individuals, lowered the number of family functions, and reduced an individual’s participation in social functions. On the other hand, the Internet may facilitate human connection with family members who live far away, reconnect with old acquaintances, and meet new people. According to Geraci et al. (2022), there was no indication that in the UK, broadband access can displace offline relationships like getting together with friends. An individual’s social life may be protected by using online networks to cope with increased business and a decrease in leisure time (Antoci, Sabatini, & Sodini, 2015). Sabatini and Sarracino (2017) found that online network participation significantly reduced trust in all its forms. The more time spent online, the more lonely an individual may feel, while it has the opposite effect on the level of happiness (Stepanikova et al. 2010). Bauernschuster et al. (2014) showed that the Internet might impact social capital. To counteract a decline in social cohesion and a weakening of links, internet access has been shown to have a positive impact on social interactions, including connectivity and the extension of social networks (Wang and Wellman 2010), reducing the disruption of connections and also reducing a diminished sense of community (Sabatini and Sarracino 2014). In light of the disparities between the findings of the aforementioned studies, the purpose of the current study is to examine the effect of internet access on social capital in Indonesia.

It is expected that this research will make a meaningful empirical contribution to the study of the effect of internet access on Indonesian social capital in all areas of Indonesia, both Java Island and others. Given that the majority of internet access in Indonesia in 2014 are concentrated on the island of Java (especially Jakarta and Surabaya) and Bali, where 52 million or 59 percent of Indonesia’s total population has access to the Internet (APJII 2015). This is not something that has been done before in previous studies. In addition, the researchers in this study looked at how women and men had access to the Internet. A policy proposal based on the findings of this research is looked for in light of today’s coronavirus epidemic, in which the Internet is being used to build social capital.

## Data and methodology

### Data

This research uses the data from the fifth wave of the Indonesian Family Life Survey (IFLS) collected by RAND Corporation and conducted at the end of 2014 to early 2015 with a sample size of 50,148 individuals and 16,204 households. IFLS5 data are specifically available for questions related to internet use or access by household members aged 15 years or older. The data are used to capture the number of respondents who use or access the Internet in Indonesia. The exploratory factor analysis method is used to calculate the social capital index. Using a scale of 10, all data scales are unified at the data preparation stage (Badan Pusat Statistik 2014). In addition, principal component analysis (PCA) is used to retrieve the formed factor. Trust, cooperation, and social networks are all markers of social capital.

- Trust
  The list of questions for the measurement variable trust is contained in Table 1.
- Cooperativeness
  The “I am willing to help people in this village if they need it” question and its original responses of 1 (strongly agree), 2 (agree), 3 (disagree), and 4 (strongly disagree) yield the cooperativeness variable. The answers will then be indexed in reverse with “Strongly agree” marked as 4 and “Strongly disagree” as 1 for this question to facilitate the index calculation.
- Social networks
  The social networks variable is obtained from the question "During the last 12 months did you participate in or use (community activities?)”. Again, to accommodate the calculation of the index the answer “yes” will be coded as one and the answer “no” will be coded 0, as opposed to the original coding which was the opposite.

**Table 1:** List of trust question

Code	Question
TR02	In this village I have to be alert or someone is likely to take advantage of me
TR03	Taking into account the diversity of ethnicities in the village, I trust people with the same ethnicity as mine more
TR04	I would be willing to leave my children with my neighbors for a few hours if I cannot bring my children with along
TR05	I would be willing to ask my neighbors to look after their house if I leave for a few days?
TR06	How safe do you consider this village?
TR07	In most parts of the village, is it safe for you to walk alone at night?
TR23	Taking into account the diversity of religions in the village, I trust people with thesame religion as mine more
TR24	How do you feel if someone with different faith from you lives in your village?
TR25	How do you feel if someone with different faith from you lives in your neighborhood?
TR26	How do you feel if someone with different faith from you rent a room from you?

Source: IFLS 2014

### Estimation strategy

This research used econometrics method to see the effect between internet access and social capital in Indonesia. To anticipate the occurrence of omitted variable bias due to the dependent variables that correlate with errors, this research also used an instrumental variable model. Therefore, a new variable is needed as an instrument as long as variable (z) does not correlate with error ($u$) or cov (z, $u$) = 0, and variable (z) correlates with the dependent variable ($x$) or Cov (z, $x$) ≠ 0 (Wooldridge 2012).

The estimation model in this research is done by applying the framework that has been done (Bauernschuster et al. 2014) Sabatini and Sarracino 2017) with some modifications. The basic model used in this study is as follows:

1 $${SC}_{i}= \alpha +{\beta }_{1}{I}_{i}+{\beta }_{2}{X}_{i}+{\beta }_{3}{Y}_{i}+{\beta }_{4}{Z}_{i}+{\varepsilon }_{i},$$

in which ${SC}_{i}$ is the social capital, ${I}_{i}$ is internet access by individuals, ${X}_{i}$ is an individual characteristic vector, ${Y}_{i}$ is a household characteristic, ${Z}_{i}$ is a community characteristic and ${\varepsilon }_{i}$ is an error term. Equation (1) shows a two-way causal relationship between internet access and social capital, which raises the problem of endogeneity bias. Due to the endogeneity problem, the ordinary least squares method is not appropriate because endogenous variables are correlated with disturbance. Applying the OLS model without correcting endogeneity will cause an estimator to be biased and inconsistent, resulting in incorrect conclusions. For example, selection bias occurs in individuals who are shy and lonely in nature, who access the Internet as they have less social contact in the real world and consequently seek compensation in the virtual world. Another example is reverse causality which may arise if individuals are politically interested. They are more likely to buy internet access because they want to access the Internet to obtain more information about politics. Another issue is the omitted variables, which are another source of endogeneity; for example, outgoing and open-minded individuals tend to socialize more, but at the same time, they are more vulnerable to new technology development such as the Internet. Thus, to overcome this problem, the instrumental variable (IV) method is carried out to overcome the bias problem. Therefore, Eq. (1) becomes as follows:

2 $${SC}_{i}= \alpha +{\beta }_{1}{I}_{i}+{\beta }_{2}{X}_{i}+{\beta }_{3}{Y}_{i}+{\beta }_{4}{Z}_{i}+{\varepsilon }_{i},$$

3 $${I}_{i}=\eta + {{\delta }_{1}\rho }_{i}+{{\delta }_{2}X}_{i}+{\delta }_{3}{Y}_{i}+{\delta }_{4}{Z}_{i}+{\mu }_{i}.$$

${\rho }_{i}$ is an instrument variable and ${\mu }_{i}$ is an error term. The instrument variables used are expected to correlate with internet access and are not related to error term. The instrument variable candidates in this research are: location/media to access the Internet at home and access to a telephone (Bauernschuster et al. 2014). The instrument variables candidates will be analyzed for their strength and validity as instruments forming internet use by assumptions that are correlated with internet use but not correlated with errors.

## Results and discussion

Table 2 reveals that Indonesians average number of internet access during the 2014 survey period was 29.4 percent, indicating a low rate. This is partly because internet access in Indonesia are not evenly spread geographically. Most internet users in Indonesia are located in Western Indonesia, such as Java (especially in big cities like Jakarta and Surabaya), Bali, and Sumatra (APJII 2015).

**Table 2:** Descriptive statistics

Variable	Obs	Mean	Std.Dev
Internet access (yes = 1)	16782	0.294	0.456
Age of household head (years)	16782	40.409	16.295
Household size	16782	4.179	1.857
Household size squared	16782	20.911	19.919
Household head education			
No school	16782	0.072	0.260
Elementary school	16782	0.449	0.497
Junior high school	16782	0.148	0.355
High school	16782	0.207	0.405
University	16782	0.081	0.273
Gender of household head (male = 1)	16782	0.859	0.349
Marital status of household head (married = 1)	16782	0.854	0.353
Employment status of household head (working = 1)	16782	0.877	0.328
Income (log)	16782	923391	826551
Homeownership status (self-owned = 1)	16782	0.844	0.363
Household utilize electricity (yes = 1)	16782	0.994	0.080
Well main water source (yes = 1)	16782	0.426	0.495
The main kind of fire/stove used for cooking is firewood (yes = 1)	16782	0.266	0.442
Type of residence (rural areas = 1)	16782	0.463	0.499
Access credit from non-financial institutions (yes = 1)	16782	0.829	0.377
Access to village credit institutions (yes = 1)	16782	0.358	0.479
Asphalt road in the community (yes = 1)	16782	0.920	0.272
Village use system of sewage channels/gutters (yes = 1)	16782	0.652	0.476

Source: IFLS 2014

The average age of head of households in Indonesia in 2014 was dominated by the productive age of 40 years, and they had four family members. In general, heads of households are primary school graduates, reaching 44.9 percent, and is dominated by men (85.9 percent) with married status reaching 85.4 percent. Most of them are workers (87.7 percent) with an average monthly income of Rp.923,391.00. Households who own homes are quite high, reaching 84.4 percent, while those with no homeownership are at 15.6 percent, most households have a power connection (99.4 percent). Households that use wells for drinking is quite high (42.6 percent), while households that burn wood to cook is quite low (26.6 percent).

Furthermore, most Indonesians live in urban areas reaching 53.7 percent, while the rest in rural areas amounted to 46.3 percent. Households with access to credit from non-financial institutions are very high at 82.9 percent. In comparison, those that access the Village Credit Institution (*Lembaga Kredit Desa*) are still low (35.8 percent). This indicates that households in Indonesia in the 2014 survey period were generally more likely to go to non-financial institutions such as village-level commodity middlemen rather than to banks to obtain a loan, even though, the middlemen charged higher interest rates than banks. This is especially apparent in rural areas where the majority engage in agricultural activities. Villages show that the main road infrastructure with asphalt in rural areas has reached 92 percent. The average availability of system of sewage channels/gutters in the village is quite high, at 65.2 percent, indicating an even availability of system of sewage channels/gutters in the village.

Instrument variables in this research are: the location/media to access the Internet at home and the distance to the location of telephone facilities (Bauernschuster et al. 2014). The instrument variables used are expected to correlate with internet access and are not related to error term. If all the independent variables entered into the model are exogenous in the sense that it does not correlate with errors, there will be no difference in the estimation results in the OLS and IV models. Therefore, it is imperative to know whether those independent variables are endogenous or not.

To see the under-identification test, the researcher used Kleibergen–Paap rk LM statistics. The estimation results of all models show that the null hypothesis of under-identification is rejected. This means that the instrument variables used are relevant, and the parameters have been correctly identified, where the variable of internet access at home and distance to telephone facilities are good instruments. Next, to test the strength of the instrument, the Cragg-Donald F-statistics is used. The Cragg-Donald F Statistics test results show a value higher than the critical value, indicating the instrument variables used in all models to be strong. Finally, for over-identification testing, the Hansen J statistic test is performed. The test results show that all instruments used in the study are exogenous.

### Internet access and social capital

Table 3 compares the estimation results and demonstrates that the IV model’s coefficient is bigger than the OLS model’s. In this study, it was found that internet access is an exogenous factor in social capital. If reverse causality is present in a 2SLS regression, the social capital coefficient value should be smaller than the OLS. Bauernschuster et al. (2014) state that the coefficient IV is greater than OLS because of the possibility that a downward bias could dominate an upward bias. People who tend to avoid real-world social contacts tend to prefer internet access. Thus, choosing to subscribe to the Internet can indirectly reduce and even be disconnected from the community. Moreover, OLS estimates may suffer from attenuation bias due to measurement error. Lastly, the causal effect of internet access on social capital may differ between the total population and subgroups due to the unavailability of technology; for example, the western part of Indonesia and the eastern part of Indonesia have differences in infrastructure on internet technology. Since the IV estimate is unaffected by the measurement error in the treatment variable, they tend to be larger than the OLS estimates (Becker 2016). Therefore, the IV model is more appropriate than the OLS model for the case in this research.

**Table 3:** Internet access and social capital

Variables	OLS	IV
	(1)	(2)
Internet access (yes = 1)	–0.013*** (0.003)	0.037** (0.017)
Household characteristics		
Income (log)	0.001*** (0.000)	0.002*** (0.000)
Age of household head (years)	0.000*** (0.000)	0.001*** (0.000)
Household size	0.004*** (0.002)	0.005*** (0.002)
Household size squared	−0.000*** (0.000)	-0.001*** (0.000)
Marital status of household head (married = 1)	0.013*** (0.004)	0.017*** (0.004)
Household head education		
Elementary school	0.022*** (0.003)	0.021*** (0.003)
Junior high school	0.030*** (0.004)	0.029*** (0.004)
High school	0.031*** (0.003)	0.026*** (0.004)
University	0.040*** (0.004)	0.023*** (0.007)
Employment status of household head (working = 1)	0.022*** (0.003)	0.026*** (0.003)
Homeownership status (self-owned = 1)	0.020*** (0.003)	0.018*** (0.003)
Fire/stove used for cooking is firewood (yes = 1)	0.006*** (0.002)	0.008***(0.002)
Well main water source (yes = 1)	0.006*** (0.002)	0.007*** (0.002)
Household utilize electricity (yes = 1)	0.001 (0.011)	−0.003 (0.012)
Community characteristics		
Type of residence (rural areas = 1)	0.012*** (0.002)	0.017*** (0.003)
Village use system of sewage channels/gutters (yes = 1)	−0.002 (0.002)	−0.003 (0.002)
Access credit from non-financial institutions (yes = 1)	0.013*** (0.002)	0.013*** (0.002)
Asphalt road in the community (yes = 1)	−0.004 (0.004)	−0.005 (0.004)
Access to village credit institutions (yes = 1)	0.008*** (0.002)	0.008*** (0.002)
Constant	0.385*** (0.014)	0.334*** (0.023)
*N*	16782	16782
Kleibergen–Paap rk LM statistic		158.486***
Cragg-Donald F statistic		484.498***
Hansen J statistic		0.2599

Source: Author

Robust standard errors in brackets, *p < 0.1, **p < 0.05, ***p < 0.01

The results of the Kleibergen–Paap LM statistic on under-identification are statistically significant, which provides evidence to reject the null hypothesis about the under-identification of the instrument. The same results on the Cragg-Donald F Statistics test are statistically significant, indicating that the instrument is not weak. Moreover, the Hansen J statistic in the model is not significant and implies that we failed to reject the null hypothesis. Thus, the instrument used in this research is valid (i.e., not correlated with the error term). Based on all these results, the instrument is valid, not weak and relevant, and thus the estimated model is reliable.

The instrumental variable (IV) estimation results showed that internet access positively affects social capital and is statistically significant at the 95% level. The premise of cateris paribus shows that if someone uses the Internet, their social capital will rise by 0.037 points. According to this study’s findings, people’s social capital appears to rise in direct proportion to the size of their online communication network. Despite the lack of face-to-face connection, past research has shown that online activities contribute positively to the development of social capital, and the final discussion examines prospective research paths for tracing causal mechanisms in the digital age of social capital formation (Hooghe and Oser 2015). The Internet is fundamentally different from television in that it delivers active information and communication services that directly impact social capital. According to others, the Internet’s primary role in social engagement is to facilitate active information and conversation, which the Internet gives in a personalized form at any time. According to this perspective, most individuals access the Internet for information and communication purposes, whereas a small percentage of people access it solely for entertainment purposes (Bauernschuster et al. 2014). As a result, the Internet serves as a means of communication and information exchange. Now, people may meet and develop new kinds of communities over the Internet; they can also sustain old ones. According to Pénard and Poussing (2010), the Internet can improve social capital by removing distances between people and allowing them to communicate with each other through mediated communication over the Internet. Additionally, according to other research, internet access has been shown to boost social capital and consolidate previously acquired social advantages (Neves et al. 2018). Even more importantly, the Internet can help people overcome social anxiety and negative feelings linked with loneliness by allowing them to connect with others online (Clayton et al. 2013).

The relationship between household income and social capital is statistically significant. According to this study, people with higher incomes are more likely to participate in social groups and view their participation as a form of leisure or luxury consumption (Christoforou 2011). In addition, wealthy individuals may consider setting up a safety net (insurance) for themselves by investing a portion of their earnings in community service or other forms of collective social capital to maximize their social capital’s return. According to past studies, personal relationships, social network support, civic involvement, and trust and cooperative norms are all affected by household income (Masato, Makoto, and Yodo 2017). Personal relationships and trust, as well as cooperative norms, may have an impact on household income, according to (Growiec and Growiec 2016).

The social capital increases with the average age of its members, and this effect is statistically significant. As earlier studies have shown, age is a factor of social capital, so older people are urged to join social groups or organizations. Personal traits such as gender, age, ethnicity, and parental (or other family members) attitudes and behaviors influence social capital, as they are likely to create the group’s norms and values (Morgan 2010). It has been found that persons who spend more than two hours a day on the Internet are also the most gregarious people in the real world (Hooghe and Oser 2015). As people get older, they lose social capital, but this varies by internet user type. While older persons were less likely to have high levels of social capital, regular internet users had greater levels than other internet users and non-users within this age range. It appears that the Internet helps people keep and build their social capital. However, social disparity and cumulative advantage may also be reinforced (Neves et al. 2018).

Household size has a positive effect on social capital and is statistically significant. Kaasa (2013) found that households with more members tend to have more social capital than those with fewer members, mainly because they play an important role in activities with mutual help. Individuals with large households tend to participate in microcredit groups as they have a family burden in terms of social and economic services, and they need support to meet their family’s daily needs (Kangogo, Lagat, and Ithinji 2013).

When it comes to building social capital, married head of household couples have a positive effect (statistically significant). Specifically, women are more inclined to engage in social capital activities motivated by economic considerations when married. Since she is now responsible for her own household’s upkeep, a married woman is more inclined to partake in social events. In addition, women can contribute to social capital activities by using their husband’s money to raise the family’s living standard even higher. Married household heads are more likely to engage in social activities because they play an essential role in elevating the social standing of the household. These findings follow previous research showing that being a parent has a favorable impact on social capital quality for married people but a negative one for single people (Song 2012).

The education of the head of households from elementary to university level has a positive effect on social capital and is statistically significant. A household head’s education level has a significant effect on his or her participation in social capital activities. According to prior studies, schools are more effective than families at promoting social capital in the community (Aghion et al. 2010). Individuals with a higher level of education invest more in social capital but receive a bigger return. Therefore, those with a higher degree of education are more willing to invest in social capital because the returns are larger (Jordan et al. 2010). Therefore, the culture of social participation is higher the better the educational outcomes and the educational settings are.

Employed head of households have a positive effect on social capital and are statistically significant. This suggests that the working head of household has a role in enhancing social capital bonding activities, such as family, relatives, and friends being able to convey useful information about work. Head of households who does not work typically devote more time to other activities, such as spending more time at home, compared to community participation or socialization. When the head of the household is unemployed, his income will be low, preventing him from setting aside funds for involvement in social capital activities. These results are consistent with earlier research indicating that unemployment tends to diminish interaction and social (Kunze and Suppa 2017).

The same thing is that homeownership contributes to social capital positively and statistically significant. This finding follows previous research that homeownership alone provides incentives for individuals to improve their communities (Roskruge et al. 2013). Moreover, people who own their own homes report much higher social capital levels than those who do not. They are more trusting of people, more involved in their local communities, and have a more positive outlook on life. Bloze and Skak (2015) said that several social capital metrics, primarily those that evaluate local involvement, are positively associated with homeownership. Previous research has shown that homeowners had more social capital and neighborhood social capital resources than renters. These data confirm this conclusion. Involvement in neighborhood groups indirectly impacts social capital, although it only accounts for a small portion of homeownership’s influence Manturuk et al. (2010).

There is a statistically significant effect between wood as the main type of fire/stove used for cooking and social capital. This finding indicates that firewood as a cooking instrument is considered an inferior good, mainly adopted in rural areas with high social capital ties. Agurto Adrianzén (2014) shows that village-level technology usage patterns (including using firewood) and bonding social capital mutually influence the individual usage decision of an improved stove in the Peruvian Andes. These findings are in line with previous research, which showed that community involvement in supporting alternative water sources is quite high, and this effect is mediated by (i) stronger social norms related to water, (ii) greater water-related knowledge, and (iii) increased withdrawal of information related to water. Thus, social capital is significant in increasing involvement in water-related issues, mainly growing support for alternative water sources (Dean et al. 2016).

Earlier studies have shown that rural places have more social capital that binds people together, such as strong social bonds and regular family-based social interactions (Sørensen 2016). Qin et al. (2022) that Japan’s urbanization and population decline have changed people’s behavior and increased their desire to connect with others in social networks, increasing rural Japan’s bridging social capital. Thus, it is believed that rural communities will continue to maintain cultural sustainability by upholding local cultural norms and values, which can play an essential role in increasing the social capital index in rural areas.

Non-financial institutions and Village Credit Institutions (LKD) have a beneficial influence on social capital and are statistically significant in terms of social capital. The findings show that access to credit from non-financial institutions was more affected than the Village Credit Institution (LKD), as it is easier to go through the credit process in the non-formal financial institutions. Non-formal financial institutions have no collateral requirements, and it has a fast loan disbursement process. Research has shown that those who have access to credit are more likely to have higher levels of social capital, which is consistent with this study’s findings (Postelnicu and Hermes 2018).

### Internet access and social capital based on gender

Table 4 shows the instrumental variables used are relevant, and the parameters have been correctly identified, whereas access to the Internet at home and distance to telephone facilities are good instruments. Cragg-Donald F Statistics test results have a greater value than critical the value so that the instrument variables used in all models are not weak instruments. Finally, over-identification testing indicates that all instruments used in the study are exogenous.

**Table 4:** Internet access and social capital by gender

Variables	OLS		IV	
	Male	Women	Male	Women
	(1)	(2)	(3)	(4)
Internet access (yes = 1)	–0.011*** (0.003)	–0.024*** (0.007)	0.033* (0.018)	0.079 (0.055)
Constant	0.385*** (0.016)	0.420*** (0.034)	0.340*** (0.024)	0.340*** (0.070)
*N*	14407	2375	14407	2375
Kleibergen–Paap rk LM statistic			141.514	18.384***
Cragg-Donald F statistic			448.037	43.087***
Hansen J statistic			0.5460	0.1859

Source: Author

Variable control including: income (log), age of household head (years), household size, household size squared, marital status of household head (married = 1), elementary school, junior high school, high school, university, employment status of household head (working = 1), homeownership status (self-owned = 1), fire/stove used for cooking is firewood (yes = 1), well main water source (yes = 1), household utilize electricity (yes = 1), type of residence (rural areas = 1), village use system of sewage channels/gutters (yes = 1), access credit from non-financial institutions (yes = 1), asphalt road in the community (yes = 1), access to village credit institutions (yes = 1)

Robust Standard errors in brackets, *p < 0.1, **p < 0.05, ***p < 0.01

If the head of the household is male, internet access positively influences social capital and is significant at the 90% level. As a result, the Internet significantly affects the social capital of male heads of households, especially in relationships between neighbors with a strong sense of unity and mutual trust within the organization. Thus, male heads of households tend to have significantly higher levels of participation in social groups (Christoforou 2011). As a result, men are more likely than women to engage in the vast majority of online activities. Men tend to have higher labor participation rates, consequently creating more diversity of work, and further, men with jobs requiring strength have more meetings with others. As such, men are over-represented in the network. Women are less likely to participate in online work-related communities than men. Women have fewer opportunities to contact people from various jobs (Miyata, Ikeda, & Kobayashi, 2012). Several obstacles stand in the way of more women using the Internet, including lack of familiarity, high costs of equipment and services, a lack of digital competence, concerns about online safety, and a lack of relevant information (Anandhita and Ariansyah 2018). Meanwhile, other variables, such as household and community characteristics, are consistent with previous estimates, effective, and statistically significant on social capital.

### Internet access and social capital by region

As a next step, we will look at how internet access affects Java, Bali’s social capital, and other parts of Indonesia. Table 5 shows that the instrument variables are relevant, and the parameters have been correctly identified, are not weak, and are exogenous. This is reflected in the under-identification tests, Cragg-Donald F Statistics, and over-identification. The estimation results of internet access on social capital through instrumental variables (IV) are based on the sub-sample of the Java-Bali region and outside of Java and Bali. Having access to the Internet in the Java-Bali region has a favorable influence on social capital and is statistically significant at 90% level. These findings indicate that internet access in Java and Bali contributes significantly to the development of social capital. In Java and Bali, internet availability has a significant impact on social capital due to the majority of internet users in Indonesia living in the western part of the country, especially in Java and Bali, with 36.9% of people in Java being internet users. Besides, around 83.4% of internet users in Indonesia live in urban areas (APJII 2015). This illustrates the inequality in the development of internet infrastructure in Indonesia and the availability of internet service in every region in Indonesia, despite reliable internet access in every region. Rural areas have also been identified as critical in strengthening social capital. Meanwhile, other variables are affected and statistically significant on social capital in the Java and Bali regions, consistent with the results of previous estimates.

**Table 5:** Internet access and social capital by region

Variables	OLS		IV	
	Java-Bali	Outside Java-Bali	Java-Bali	Outside Java-Bali
	(1)	(2)	(3)	(4)
Internet access (yes = 1)	−0.018*** (0.003)	−0.006 (0.004)	0.040* (0.023)	0.036 (0.025)
Constant	0.388*** (0.027)	0.404*** (0.018)	0.325*** (0.037)	0.364*** (0.031)
N	14407	2375	14407	2375
Kleibergen–Paap rk LM statistic			84.759	81.379***
Cragg-Donald F statistic			239.462	275.241***
Hansen J statistic			0.5581	0.1779

Source: Author

Variable control including: income (log), gender of household head (male = 1), age of household head (years), household size, household size squared, marital status of household head (married = 1), elementary school, junior high school, high school, university, employment status of household head (working = 1), homeownership status (self-owned = 1), fire/stove used for cooking is firewood (yes = 1), well main water source (yes = 1), household utilize electricity (yes = 1), type of residence (rural areas = 1), village use system of sewage channels/gutters (yes = 1), access credit from non-financial institutions (yes = 1), asphalt road in the community (yes = 1), access to village credit institutions (yes = 1)

Robust Standard errors in brackets, *p < 0.1, **p < 0.05, ***p < 0.01

### Robustness check

In this study, the robustness check is done by adding various community characteristics related to public facilities in rural areas. In this case, the distance to follow the activity together for the immediate public interest (such as: building public facilities, community service, etc.) will indirectly affect social capital. Table 6 shows that the estimation results are consistent with the previous models, where the Internet has a positive effect on social capital and is significant at the 95 percent level by adding various community characteristics related to transportation facilities in the village.

**Table 6:** Internet access and social capital: distance to public facilities

Variables	OLS	IV
	(1)	(2)
Internet access (yes = 1)	−0.013*** (0.003)	0.036** (0.017)
Distance to public facilities		
Nearby regencies (km)	−0.015*** (0.004)	–0.017*** (0.004)
Nearest districts (km)	−0.001 (0.003)	0.000 (0.003)
Nearby terminals (km)	0.009** (0.004)	0.010** (0.004)
Nearest post office (km)	0.004 (0.003)	0.005 (0.003)
Nearest terminal for vehicles with 3 wheels (km)	0.010*** (0.004)	0.010*** (0.004)
Nearest markets (km)	−0.003 (0.003)	−0.002 (0.003)
Constant	0.383*** (0.014)	0.333*** (0.023)
*N*	16782	16782
Kleibergen–Paap rk LM statistic		157.319***
Cragg-Donald F statistic		474.335***
Hansen J statistic		0.2137

Source: Author

Variable control including: income (log), age of household head (years), household size, household size squared, marital status of household head (married = 1), elementary school, junior high school, high school, university, employment status of household head (working = 1), homeownership status (self-owned = 1), fire/stove used for cooking is firewood (yes = 1), well main water source (yes = 1), household utilize electricity (yes = 1), type of residence (rural areas = 1), village use system of sewage channels/gutters (yes = 1), access credit from non-financial institutions (yes = 1), asphalt road in the community (yes = 1), access to village credit institutions (yes = 1)

Robust Standard errors in brackets, * p < 0.1, ** p < 0.05, *** p < 0.01

## Conclusions

Three findings can be concluded from this research. First, the high internet access in Indonesia plays an essential role in strengthening social capital. Social capital is an individual attribute that allows people to utilize the resources of other members in their location to obtain greater monetary and non-monetary benefits from social interactions, such as valuable information, better working conditions and lives, better social status, happiness, or self-esteem. Second, internet access plays a significant role in strengthening social capital, particularly for male heads of households with high internal cohesion and mutual trust with their neighbors. Men tend to have higher labor force participation rates resulting in more diversity of work. Moreover, men tend to meet more people in their work. As such, men are over-represented in networking. On the other hand, women are less likely to participate in online work-related communities than men, so they have fewer opportunity to interact with individuals from other occupations. Third, Internet access has a significant impact on social capital in Java and Bali since the bulk of internet users in Indonesia reside in the western portion of the country, particularly in Java and Bali. In addition, there are disparities in the development of Indonesia’s internet infrastructure and the availability of internet connection services around the country. This study contains limitations due to the use of cross-sectional data. Thus, it is hoped that future research on internet access and social capital will include longitudinal data to compensate for the possibility of unobserved variation that can influence outcomes. Future research may take age dynamics into account when examining the impact of technology on social capital and the growth of digital technology on social stratification. In addition, it takes into account psychosocial competencies that are anticipated to enhance comprehension of the relationship between Internet access and social capital in old age. Lastly, this study does not yet distinguish the impact of internet access on particular social capital functions (for example, bonding, bridging and linking). Therefore, future research may examine the diverse roles of social capital in relation to Internet access in Indonesia.

## Data Availability

Available.
